# Neuroimaging results suggest the role of prediction in cross-domain priming

**DOI:** 10.1038/s41598-018-28696-0

**Published:** 2018-07-09

**Authors:** Catarina Amado, Petra Kovács, Rebecca Mayer, Géza Gergely Ambrus, Sabrina Trapp, Gyula Kovács

**Affiliations:** 1 Biological Psychology and Cognitive Neurosciences, Institute of Psychology, Friedrich Schiller University Jena, 07743 Jena, Germany; 2 Brain Imaging Centre, Research Centre for Natural Sciences, Hungarian Academy of Sciences, 1117 Budapest, Hungary; 3 Laboratory of Cognitive Neuroscience, Department of Psychology, Theodor-W.-Adorno-Platz 6, D-60323 Frankfurt am Main, Germany; 4 Cognitive Psychology, Faculty of Psychology and Human Movement Science, University of Hamburg, 20146 Hamburg, Germany

## Abstract

The repetition of a stimulus leads to shorter reaction times as well as to the reduction of neural activity. Previous encounters with closely related stimuli (primes) also lead to faster and often to more accurate processing of subsequent stimuli (targets). For instance, if the prime is a name, and the target is a face, the recognition of a persons’ face is facilitated by prior presentation of his/her name. A possible explanation for this phenomenon is that the prime allows predicting the occurrence of the target. To the best of our knowledge, so far, no study tested the neural correlates of such cross-domain priming with fMRI. To fill this gap, here we used names of famous persons as primes, and congruent or incongruent faces as targets. We found that congruent primes not only reduced RT, but also lowered the BOLD signal in bilateral fusiform (FFA) and occipital (OFA) face areas. This suggests that semantic information affects not only behavioral performance, but also neural responses in relatively early processing stages of the occipito-temporal cortex. We interpret our results in the framework of predictive coding theories.

## Introduction

The repeated presentation of a given stimulus leads to several behavioral and neural consequences. On the one hand, stimulus repetitions enhance performance, as indicated by shorter reaction times, an effect referred to as (immediate) repetition priming^1,2^. On the other hand, both macaque and human experiments have shown that stimulus repetition reduces single-cell activity^3,4^, the amplitude of event-related potential (ERP) components^5^, as well as the magnitude of the blood-oxygen level dependent (BOLD) signal in functional magnetic resonance imaging (fMRI) experiments^6^. This response reduction is typically referred to as repetition suppression (RS) while in the neuroimaging literature, it is often referred to as fMRI adaptation (fMRIa^7^).

Recently, the phenomena of RS and fMRI adaptation (fMRIa) have been connected to the process of stimulus predictions. According to the predictive coding framework, feedback connections convey prior hypotheses about the environment, and these are compared with feedforward connections by estimating an error signal, i.e., a mismatch between the predicted hypothesis and the actual sensory input^8^. The error signal equals to the amount of sensory evidence that cannot be “explained away” by the hypothesis. This prediction error (PE) is used to fine-tune the internal generative model through an iterative process, which occurs until the PE is eliminated. Finally, this process results in models which optimally represent the causes of sensory stimuli. Consequently, surprising/incorrectly predicted events generate larger neural activity in comparison with correctly predicted events, maximizing the efficiency of neuronal processing^9–11^. Given the stability of our visual environment (scenes and objects are usually constant, and change only relatively rarely^12^), stimulus repetition may be encoded as a ‘default’ prior (i.e. as the most fundamental form of predictions^13^) in the brain. Indeed, direct evidence that fMRIa is a consequence of the ‘default’ prior of repetition has been found when predictions are modulated by statistical probabilities^14–19^ or by explicit cues^20,21^. Specifically, it has been suggested that the repetition of a stimulus reduces the mismatch or PE between the expected and the actual incoming stimulus, and that it is this error reduction that is manifested in fMRIa (for a review see^22^). As of today, it is still unknown if and how fMRIa and the repetition priming effects, as observed in behavior, are related to each other. For example, it was suggested that repetition priming and fMRIa have different neural backgrounds^23^, also a dissociation of the two phenomena was found, depending on the interval between prime and target^24^.

Importantly, performance enhancements are not limited to the mere repetition of the same stimulus: previous encounters with similar, closely related primes also lead to faster or more accurate processing of subsequent targets (referred to as semantic or associative priming^25^). If the prime and target originate in different modalities (e.g., acoustic and visual), or in different stimulus domains (e.g., written text and visually presented shape), the effect is often referred to as cross-domain priming^26^. For example, when the prime is a name, and the target is a face^25,27,28^, the recognition of a familiar persons’ face is facilitated by the prior presentation of its name or by that of a related familiar person^29,30^.

Interestingly, so far only a few studies tested the neural background of such cross-domain behavioral priming effects and their commonalities with neural RS. As of yet, the electrophysiological data available suggests that cross-domain arrangements lead to weaker effects than domain-specific priming, and seem to occur at later stages of processing, i.e., corresponding to the N400 component in event-related potentials^29–33^. Interestingly, there is neuroimaging evidence that prior auditory information affected the visual processing of objects in the left fusiform gyrus^34^. Briefly, this region was found to be sensitive to the repetition of a given object when its name was visually or auditorily presented.

To the best of our knowledge, so far, no study used fMRI to probe the neural correlates of cross-domain effects with names priming faces. Here, we conjecture that the effect of cross-domain priming is comparable to the effects reported in predictive cueing arrangements, which have been broadly tested in fMRI experiments^20,21,35–39^. In such experiments, a neutral stimulus (the cue) is paired with a subsequent stimulus via learning and can, thereby, evoke perceptual expectations. The main finding of these studies is that valid cues lead to behavioral facilitations as well as to reduced BOLD responses in comparison to invalid cues. For example^38^, used red/blue/green lines as cues which predicted the orientation of subsequent Gabor patches and found that the BOLD signal in the middle occipital gyrus was reduced for valid as compared to invalid trials. Egner and colleagues^37^ associated green/blue frames with the high likelihood of face/house presentations. They found that valid cues reduced the activity of the fusiform face area (FFA) and the parahippocampal place area when compared to the invalid trials. The authors interpreted their results in the predictive coding framework and argued that feature expectation and surprise determine the visual responses more than the input, i.e., the features per se. In an fMRI study by^39^, letters served as predictors for faces, and the BOLD activity in the FFA was modulated as a function of the probability with which the cue was associated with the face. Another fMRI study by^36^, tested cross-domain priming effects through name-object associations where the names could be congruent or incongruent with a degraded and gradually revealed visual object. They found that object recognition occurred earlier for congruently than for incongruently primed objects and that the BOLD signal of the fusiform cortex was modulated by the degradation level of the stimuli. Briefly, clear stimuli induced larger neural activity for the incongruent name-object associations when compared to the congruent ones, while objects with more degradation lead to larger BOLD responses for congruently than for incongruently primed sequences^36^.

Here we reasoned that if cross-domain priming, similarly to these above mentioned cueing arrangements, leads to facilitated recognition of targets due to predictive processes^40,41^, then we should also observe fMRIa in functionally selective cortical regions for congruently primed conditions. Indeed, a recent study^42^ suggests that expectation can modulate repetition priming effects by expecting the repetition of a given stimulus. Here, we used names of famous persons as primes and their faces as targets in a familiarity task, while unfamiliar faces served as control stimuli. We expected that if predictions are made across domains, then we should observe fMRIa in the occipito-temporal face sensitive areas, similarly to the mere repetition of stimuli.

To anticipate our results, we indeed found that congruent primes led to lower BOLD signal in bilateral FFA and in the occipital face area (OFA). Altogether, our results suggest that semantic information, such as the names of individuals, also affect the neural responses in stimulus sensitive regions, such as OFA and FFA. We interpret our results in the predictive coding framework.

## Results

### Behavioural

**Figure 1:**
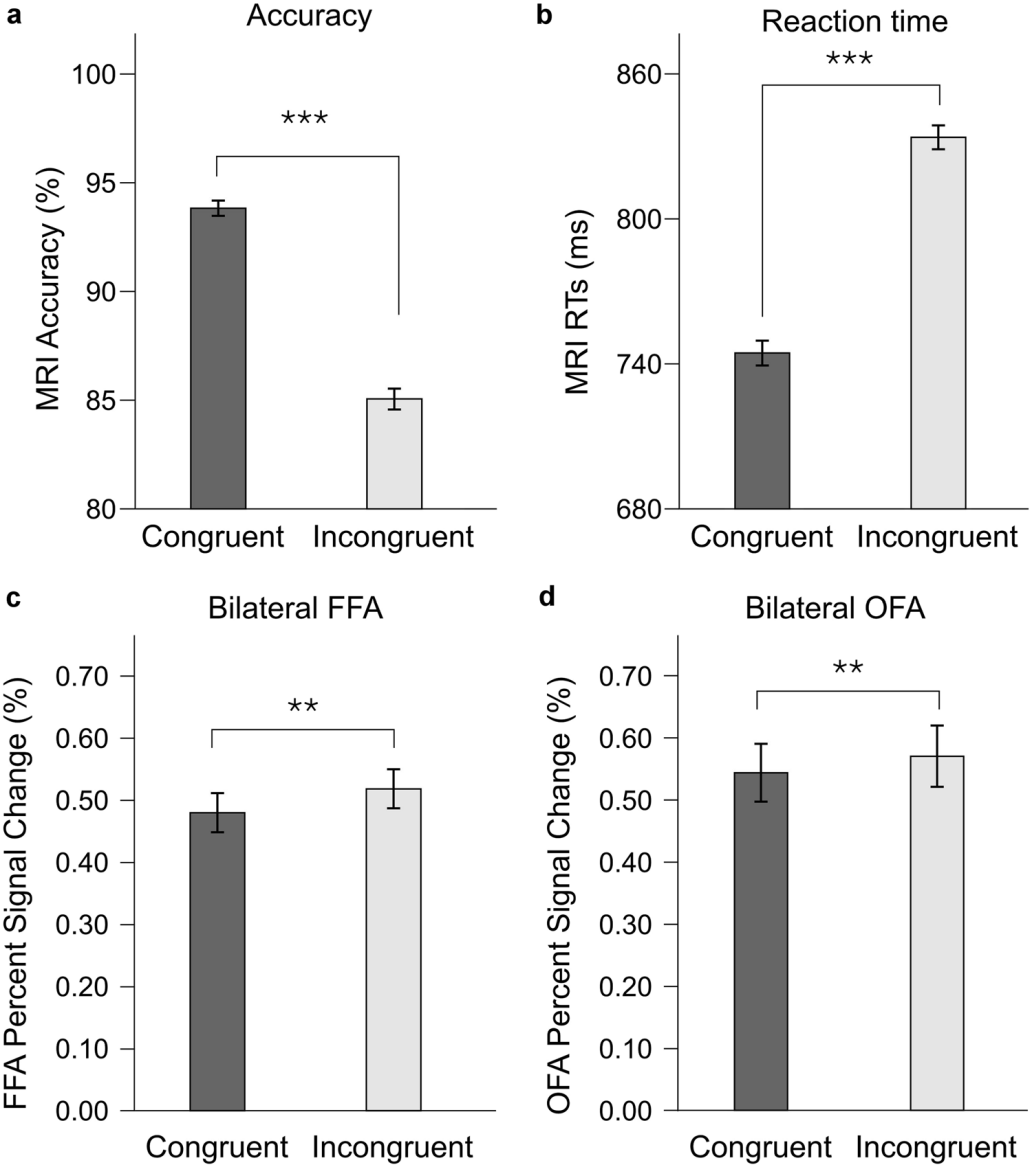
Behavioral and neuroimaging results: Mean Accuracy ((**a**) top left); Reaction Time ((**b**) top right); Percent-signal changes (±SE) are presented separately for the bilateral FFA ((**c**) down left) and for the bilateral OFA ((**d**) down right), separately for the two experimental conditions of interest (*Congruent* and *Incongruent*). ***p < 0.001, **p < 0.01.

Participants needed, on average, 789 ms (±SD: 22 ms) to determine the familiarity of the presented target face. We found a strong priming effect (main effect of condition; F(1, 17) = 50.5, p < 0.000001, ηp² = 0.75; see Fig. 1a), in other words responses were faster in the *Congruent* as compared to the *Incongruent* condition.

Mean accuracy for familiarity judgment was 89% (±SD: 0.02%) across the two experimental conditions (Fig. 1a). Similarly to the reaction time (RT) results, we found a main effect of condition on accuracy as well (F(1, 17) = 26.16, p = 0.00009, ηp² = 0.61). The priming effect (i.e. main effect of condition) was due to a reduced accuracy in the *Incongruent* condition when compared with the *Congruent* one.

### BOLD response

We found a main effect of condition in the FFA (F(1, 17) = 14.69, p < 0.01, ηp² = 0.46) and OFA (F(1, 17) = 8.77, p < 0.01, ηp² = 0.34) (Fig. 1b) in a way that the BOLD response was larger for the *Incongruent* as compared to the *Congruent* condition (p = 0.001 and p = 0.008; with an average signal reduction of 0.04% and 0.03%, corresponding to a relative signal reduction of 8% and 6%, for the FFA and OFA, respectively). These results suggest that cross-domain priming leads to a signal reduction in face-responsive areas, similar to the fMRIa observed for repeated face stimuli. Interestingly, there was a significant interaction between hemisphere and condition in the OFA (F(1, 17) = 4.71, p < 0.05, ηp² = 0.22), but not in the FFA (F(1, 17) = 0.47, p > 0.05, ηp² = 0.02). The post-hoc analysis of this OFA hemisphere x condition interaction revealed that the signal reduction is somewhat lower for the left OFA but nonetheless significant for both the right (p < 0.0001; average signal reduction of 0.05% and relative signal reduction of 9%) and the left (p = 0.03; average signal reduction of 0.02% and relative signal reduction of 4%) hemispheres. No other significant effects were found, as there was no main effect of hemisphere for both FFA (F(1, 17) = 1.9, p = 0.2, ηp² = 0.09) and OFA (F(1, 17) = .3, p = 0.6, ηp² = 0.02).

### Whole-brain

It is possible that cross-domain priming effects also occur elsewhere in the brain. Therefore, we performed a second level, whole-brain analysis testing for a main effect of condition, using a fixed threshold of p < 0.05_FWE_, with a cluster size >50 voxels. The main effect of priming (*Incongruent* > *Congruent*) revealed a significant cluster of activation in the supplementary motor cortex (SMC; MNI[x, y, z]: -6, 12, 48; cluster size: 180 voxels). To confirm that no other region remained unnoticed by the commonly applied, but rather rigorous, FWE corrected threshold we also analyzed our data at a less conservative threshold (p < 0.0001_uncorrected_; cluster extent of >20 voxels). The *Incongruent* > *Congruent* contrast showed another significant cluster in the insula (MNI[x, y, z]: −42, 8, 6; cluster size: 28 voxels). Moreover, it is possible that the differences in brain activation may reflect changes in behaviour. Thus, differences in reaction times between the two conditions of interest (i.e. congruent and incongruent) were used as a covariate in a regression analysis. Still, no region showed a correlation between the behavioural priming and the BOLD signal reduction.

### Correlation analysis of the behavioural and neuroimaging datasets

To test whether the response difference of congruent and incongruent trials is related between the behavioural and neuroimaging data, a correlation analysis was performed between the two data sets for each ROI and experimental run, separately (between and within subjects in a trial-by-trial analysis). No correlation was found between the BOLD response reduction and the behavioural priming effects (between and within subjects in a trial-by-trial analysis).

## Discussion

In the current experiment, we tested prediction effects across domains driven by name-face associations. Briefly, we reasoned that if cross-domain priming induces predictive processing for congruent prime-target trials^40,41^, then the fMRI responses of occipito-temporal face sensitive areas should be reduced for Congruent when compared to Incongruent conditions. Our major finding is that faces of familiar persons, seen after Congruent names, led to significantly lower fMRI signal in FFA and OFA. Overall, these results clearly show that the neural activity of FFA and OFA is modulated by prior semantic information, just as these regions are affected by previously presented abstract cues^20,37,39^.

Several studies suggest that fMRIa is the neuronal correlate of the behaviourally observed short-term repetition priming effects^43–45^. Indeed, short-term repetition priming effects and fMRIa have strong similarities: Both are facilitative processes induced by identical paradigms, do not depend on retrieval, and occur with different stimulus attributes^41^. Yet, whether fMRIa is a neurophysiological index of priming is still under heavy debate, as there are also evidences of the dissociation of the two phenomena^23,24^; for a review see^46,47^. It is worth noting, however, that even some of the studies which report a dissociation between fMRIa and short-term repetition priming effects show reduced neural activity for primed as compared to unprimed conditions^23,24^. Interestingly, one of these studies manipulated short-term repetition priming and fMRIa orthogonally^23^. In two sessions, subjects were given pairs of stimuli that could either repeat or alternate. Some of the pairs presented had been shown in a prior session (old pairs), while other pairs were new. Their results showed BOLD signal differences between primed (old) and unprimed (new) conditions for the presentation of different stimulus pairs. Note, however, that in this study, priming was defined having previously seen a stimulus in a separate block or not. There was a large number of intervening stimuli between primes and targets. In the current study, we measured immediate priming effects with no stimuli intervening between prime and target in order to be comparable to several previous RS studies showing prediction effects^14–17,19,21,38,48^. The existence of two kinds of fMRIa mechanisms was proposed^24^, one driven by priming processes and another one driven by pure stimulus repetitions (for a review see^49^). Indeed, as mentioned by a recent review^50^, it is suggested that fMRIa is actually influenced by both low- and high-level processing mechanisms, confirming the above mentioned hypothesis^24^.

To the best of our knowledge, the present study is the first to investigate the relationship between fMRIa and immediate cross-domain repetition priming of face perception. Our results reveal that just like fMRIa, congruent name primes lead to lower BOLD signal in bilateral FFA and OFA when compared to incongruent name primes. Such results support a study performed by Simons and colleagues^34^. This study applied cross-domain and domain-specific repetition priming effects for everyday objects and authors found that the objects could be primed by images or by their acoustically presented names. Importantly however, the authors found a huge variability across subjects in their priming effects (on the left fusiform gyrus, see figure 3 of ^34^) and, therefore, separated the participants into two sub-groups. Those differences can be explained by the fact that no separate functional localizer images were used to identify the regions of interest.

Furthermore, the findings of the current experiment are in line with existing electrophysiological studies which show a reduction of neuronal activity for cross-domain stimulus repetitions^29,30,51^. Briefly, the cross-domain priming effects of these studies were weaker, had different cortical distributions and occurred at later time-windows than domain-specific stimulus repetition effects. Although the absolute signal reduction found in FFA and right OFA in the present study is comparable to that of previous studies investigating repetition of face stimuli^20,21^, the relative signal reduction was smaller than the one reported in the above-mentioned studies. A limitation of the current experiment relies on the fact that no direct comparison was performed between such effects, i.e. repetition priming and cross-domain repetition priming (see^34^ for an example). Therefore, future neuroimaging studies are necessary to compare fMRIa and the underlying mechanisms between cross-domain and domain-specific stimulus repetitions directly, ideally within the same participants and sessions.

The finding that semantic information modulates the BOLD response in FFA and OFA cannot be explained by conventional low-level response adaptation mechanisms, as there was no stimulus repetition in our paradigm. Rather, these results suggest that semantic information is fed back to face responsive areas, presumably from the visual letter-form area (LFA^52^), visual word-form area (WFA^53–55^) or anterior and medial temporal cortices. Even though the WFA and the occipito-temporal face responsive areas have different anatomical connections, there is a considerable proportion of shared connectivity between the WFA and FFA^56^. Additionally, another study showed that the FFA is activated by the auditory stimulation of familiar identities^57^, suggesting cross-domain identity representations in this area. Furthermore, novel evidences support the idea that sensory cortices receive information from domain-specific areas (via feed-forward pathways) as well as from other sensory domains, potentially via cortical feedback connections^58,59^; for a review see^60^. However, so far, the existing literature has mostly focused on the early visual cortices and their auditory sensory inputs. These studies showed that the early visual cortices receive specific, non-retinal information by auditory stimulation and/or by imagery, which induces visual representations that can be decoded via multivariate pattern analysis^58,59^. A common critic to these findings is that the auditory-induced visual representations could occur due to memory processes in a way that the primary visual cortex is used to restore the spatial stimulus information^61^. Furthermore, none of the mentioned studies found direct evidence of behavioral facilitation due to the feedback processes in these early visual cortexes. To the best of our knowledge, the current experiment provides the first direct evidence that cross-domain connections may underlie behavioral facilitation processes.

We argue that this modulation, driven by semantic information occurs because the brain is able to ‘predict’ the occurrence of a given face if provided with a congruent name previously, and that it is this prediction that leads to reduced neural activity and processing demands. Even though there is an ongoing debate on the underlying neural mechanisms of priming, in the last years, predictive coding has been used to explain several psychophysiological and neurophysiological phenomena, such as within- and cross-domain priming effects^9,40,41^. For example, Gotts and colleagues^41^ state that repetition priming occurs through enhanced neuronal synchronization, and that it is this mechanism which leads to a firing rate reduction^62,63^. Interestingly, Pickering and colleagues^29^ found smaller ERP amplitudes of the N400 for congruent name-face associations when compared to incongruent ones, as well as a shorter latency of the N400 ERP component for the primed as compared to the unprimed conditions. This ERP latency difference between Congruent and Incongruent prime-target associations can be interpreted by the synchronization theory, which states that correctly predicted inputs lead to a synchronous coupling between selective neuronal cells and regions whereas incorrectly predicted stimuli induce their asynchronous coupling^41^. The level of synchronization between neuronal regions might represent the update of the representation units^9,10^, which are expressed simultaneously throughout the hierarchy when the sensory input is similar to the priors. However, further studies are necessary to investigate the relationship between the electrophysiological and neuroimaging data, ideally applying the same paradigm within the same participants.

Here, we propose that the underlying mechanisms of cross-domain priming are similar to the ones of short-term repetition priming and repetition suppression. We argue that cross-domain priming occurs due to the formation of predictions, presumably created by feedback, cross-connections among the domain-specific areas, which encode different aspects of the input. One may speculate that the presentation of a name will generate predictions not only in face responsive areas, but, potentially, also in the auditory cortices. Additionally, as other prediction-related processes have been shown to depend on expertise^15^, we expect that the level of multi-sensory activations depends on the experience/familiarity with the given information. In fact, a recent study shows that short-term repetition priming as well as repetition suppression are equally modulated by environmental stability^42^, in other words, when repetitions were more likely and, therefore, expected, participants exhibited greater repetition priming and repetition suppression effects than when they were less likely. Future experiments should assess whether cross-domain priming effects are also modulated by such higher-level expectations.

Furthermore, we interpret this finding as a consequence of hierarchical systems on both the face perception network and prediction related processes^22,64,65^. It is under heavy debate currently as to which extent the face processing in the OFA is limited to low-level physical features^66,67^. Recent TMS evidences show that the OFA has an important role in the encoding of face identity^65^ and in the creation of identity-specific memory traces^64^. In fact, earlier case studies have already shown that an intact rOFA is crucial for identity-dependent face processing^68,69^. Therefore, similarly to^64,65^ and to certain models of face perception^70^, we suggest that the OFA might be crucial to face recognition by playing a role in the association of visual facial and semantic information. Current anatomical studies suggest that the semantic information, probably from the WFA reaches first the FFA and thereby the OFA^56^. However, specifically designed functional connectivity and transcranial magnetic stimulation studies^71^, using cross-domain priming paradigms will reveal the hierarchy of these feedback and feedforward pathways. Nonetheless, the fMRIa during cross-domain paradigms in the OFA/FFA suggests that these areas play a role in the association of names and faces, necessary for correct person recognition.

Interestingly, no correlation was found between the neuroimaging and bahevioural results. It is possible that the neuroimaging results are a consequence of the encountered cross-domain priming effects, rather than the cause, due to different attentional levels (as participants need less time to process a congruent face and thus, might devote less attention). Although several experiments tried to disentangle this question^72–74^, for reviews see^75,76^, the involvement of attention in priming is not yet clear^77^. However, the potential attentional effects in the current experiment are relatively less likely because: 1. The face stimuli were presented for a rather short duration (of 200 ms); 2. The familiarity discrimination task could not be performed until the target face appeared on the screen. 3. Participants were informed to that a name would precede a face and that their task was exclusively related to the face rather than to the name stimuli. It is worth mentioning, however, that recent findings suggest that priming resets the on-going theta-band oscillations, which have been connected to attention and predictive coding^78^. Therefore, one potential follow-up study could test explicitly the role of the task/attention on the observed fMRI, similarly to the stimulus repetition study of^18^.

Additionally, the current experimental design can be a useful tool to investigate psychiatric disorders with deficits in predictive processing, such as autism or schizophrenia^79,80^. However, so far, there is evidence of increased^81–83^ as well as decreased priming effects in schizophrenia^83–85^. Studies that investigated priming effects in autism show behavioral facilitation processes both for healthy and ASD participants^86,87^, although there is evidence that priming effects in autism are modulated by the social context^88^. Much less is known with regard to cross-domain priming paradigms, and these may be crucial to understand to which extent prediction processes are attenuated in such neurological conditions.

In sum, our data suggest that cross-domain priming, similarly to stimulus repetitions, leads to fMRIa in the occipito-temporal cortex and that the face perception network encodes relevant semantic information in a cross-domain name-face priming paradigm. Our findings indicate that the mechanisms by which prior information facilitates a response (behaviorally or neurally) do rely on a constant and hierarchical update of predictions, supporting predictive coding theories. Furthermore, our results also suggest that the specific content of a given sensory input and its relevancy modulates the neural processes of regions which are not a priori expected to be selective to such information.

## Material and Methods

### Participants

20 healthy German volunteers (with Western cultural background) participated in the fMRI experiment (7 male; 0 left-handed, mean age (±SD): 23.39 (3.42) years; 2 subjects were excluded, one due to neurological abnormality detected after the experiment and another due to low recognition of the selected famous faces). All volunteers were informed and gave a written consent form before participating. The protocol of the experiment was approved by the Ethical Committee of the Friedrich Schiller University Jena and the experiment was performed in accordance with relevant guidelines and regulations. All participants had normal or corrected to normal vision.

### Stimuli and Procedure

**Figure 2:**
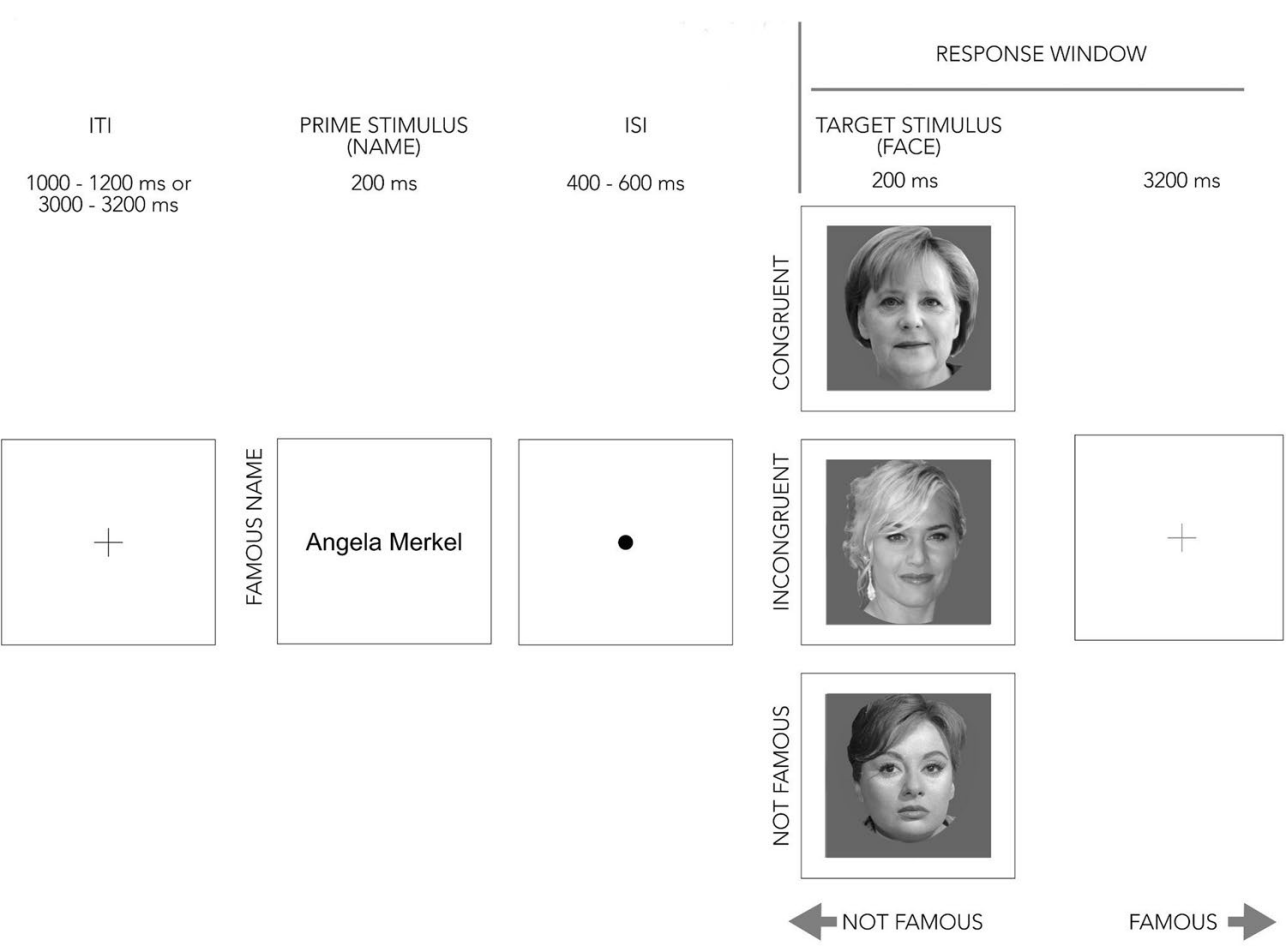
Familiarity decision task for famous and unknown target faces in the fMRI experiment. A trial began with the presentation of a fixation cross, followed by the presentation of the prime stimulus, which was a name of a famous person. Following the presentation of a fixation cross a face congruent with the prime name, a face of another famous person, or an unknown person was displayed (target stimulus). The participants were instructed to make familiarity judgements (famous/not famous) for the faces. Image credits: Congruent: Armin Linnartz [CC BY-SA 3.0 de (https://creativecommons.org/licenses/by-sa/3.0)], via Wikimedia Commons (Angela Merkel, the current German Chancellor); Incongruent: Georges Biard [CC BY-SA 3.0], via Wikimedia Commons (Kate Winslet, American actress); Not famous: Fortepan/Kotnyek Antal [CC BY-SA 3.0 (http://creativecommons.org/licenses/by-sa/3.0)], via Wikimedia Commons (Hédi Váradi, Hungarian actress). These images were all modified from the original (including grayscale conversion, background removal and resizing), and were not part of the actual stimulus set. Angel Merkel: File: Angela Merkel Juli 2010 - 3zu4.jpg. (2018, March 11). Wikimedia Commons, the free media repository. Retrieved 14:42, May 17, 2018 from https://commons.wikimedia.org/w/index.php?title = File:Angela_Merkel_Juli_2010_-_3zu4.jpg&oldid = 291809539. Kate Winslet: File: Kate Winslet César 2012.jpg. (2017, September 18). Wikimedia Commons, the free media repository. Retrieved 14:45, May 17, 2018 from https://commons.wikimedia.org/w/index.php?title = File:Kate_Winslet_C%C3%A9sar_2012.jpg&oldid = 258978664. Hédi Váradi. File: Váradi Hédi színművésznő. Fortepan 11996.jpg. (2017, October 26). Wikimedia Commons, the free media repository. Retrieved 14:46, May 17, 2018 from https://commons.wikimedia.org/w/index.php?title = File:V%C3%A1radi_H%C3%A9di_sz%C3%ADnm%C5%B1v%C3%A9szn%C5%91._Fortepan_11996.jpg&oldid = 264559848.

Two groups of visual stimuli were used: faces as targets and names as prime stimuli. The face stimulus pool included 40 familiar famous (see Table 1) and 40 unfamiliar faces (downloaded from the worldwide web). The name stimuli (Arial font with size 26 in black color) corresponded to the first and the last names of the selected familiar faces (for example: *Angela Merkel;* see Fig. 2). Thus, in total, 40 familiar names were used in this experiment. The probability of gender was equal (i.e. 50/50% female/male) for all stimulus groups. The unfamiliar identities corresponded to Hungarian celebrities, unfamiliar to the selected group of participants (with German nationality).

**Table 1:** List of famous names and faces used in the two experiments.

Male	Female
Albert Einstein	Angela Merkel
Arnold Schwarzenegger	Angelina Jolie
Daniel Craig	Anne Hathaway
David Beckham	Avril Lavigne
David Hasselhoff	Britney Spears
Elvis Presley	Cameron Diaz
George Clooney	Emma Watson
George W. Bush	Heidi Klum
Johnny Deep	Jennifer Aniston
Justin Bieber	Jennifer Lawrence
Justin Timberlake	Jennifer Lopez
Leonardo DiCaprio	Julia Roberts
Michael Jackson	Keira Knightley
Pierce Brosnan	Kristen Stewart
Prince Harry	Natalie Portman
Robbie Williams	Paris Hilton
Rowan Atkinson	Penelope Cruz
Sylvester Stallone	Sarah Jessica Parker
Tom Cruise	Scarlett Johansson
Vladimir Putin	Shakira

In order to create a pool of familiar faces, a prior behavioral experiment was performed to determine which famous identities were generally known by the German population. Identities that were correctly recognized by more than 85% of the participants were included in the final pool of familiar faces. The selected images fulfilled the following criteria: direct gaze and neutral facial expressions. The images were transformed in a way that the eyes of all faces were at the exact same position. Finally, the faces were converted to greyscale with equal contrast and luminance (SHINE toolbox^89^). Stimulus size was 3.65° in radius for the faces.

The stimuli were delivered using MATLAB R2014a (The Mathworks, Natick, MA, USA), via Psychtoolbox (Version 3.0.12) and were back-projected via an MRI-compatible LCD video projector (NEC GT 1150, NEC Deutschland GmbH, Ismaning, Germany) onto a translucent oval screen, located inside the scanner bore.

The experimental design was similar to what has been previously used by^29^ and^90^. Briefly, it consisted of a name-face cross-domain priming paradigm in which familiar, famous names primed the subsequent target faces. In the current experiment, a trial consisted of a familiar name (exposition time = 200 ms), followed by a short (400 to 600 ms with 50 ms steps) inter-stimulus interval (ISI) and a face (200 ms), which could either be unfamiliar (80 trials) or familiar (80 trials). The test faces depicted the same (*Congruent*) or a different (*Incongruent* or *Unfamiliar*) person as the prime names. In case of unfamiliar test faces the primes were also names of famous persons from our pool. Therefore, there were three conditions: *Unfamiliar*, *Congruent* and *Incongruent*. During the ISI participants saw a fixation dot. The inter-trial interval (ITI) varied between 1 s and 1.2 s (50 ms steps) or between 3 s and 3.2 s (50 ms steps). The stimulus background was always grey. Figure 2 depicts the experimental paradigm.

Importantly, the prime name itself was not predictive of the fame of the subsequent face, as it could be followed by famous or unfamous faces with equal probability. The gender of stimuli was balanced across the conditions (i.e. 50/50% male/female) and only one gender was used within a trial. In other words, the gender of the primes and targets always matched. The familiar, famous names were randomly assigned to one of the two Familiar conditions (i.e. *Congruent* or *Incongruent*) in each run.

Participants’ task was to judge whether the presented faces were familiar (i.e. famous) or unfamiliar as fast and as accurately as possible. The response time window ranged from the onset of the target stimulus until the end of the ITI.

Overall, two fMRI runs (160 trials per session) were acquired. Participants were informed that a name would precede a face and that their task was related to the face rather than the name stimuli. After the experiment, participants completed a behavioral test to assess their recognition rate of the selected familiar faces. According to these behavioral assessments, trials with false positives (i.e. when unfamiliar faces were falsely marked as famous) and misses (familiar faces that were not recognized) were removed from the statistical analysis of interest. Participants with remaining trials below 75% were also excluded from the analysis (N = 1; see^64^).

### Imaging Parameters and Data Analysis

Imaging was performed with a 3-Tesla MR scanner (Siemens MAGNETOM Prisma fit, Erlangen, Germany). Functional, T2* weighted images were collected using an EPI sequence with the following parameters: 35 slices, 10° tilted relative to axial, TR = 2000 ms; TE = 30 ms; flip angle = 90°; 64 × 64 matrices; 3 mm isotropic voxel size. A high-resolution 3D anatomical image (T1-weighted), was acquired using a MP-RAGE sequence and the parameters were the following: TR = 2300 ms; TE = 3.03 ms; 192 slices; 1 mm isotropic voxel size. Both functional and anatomical images were acquired using a 20-channel head coil. Pre-processing and statistical analysis were conducted as described in^20^ using SPM12 (Welcome Department of Imaging Neuroscience, London, UK). Briefly, the functional images were slice time corrected (the 1^st^ slice was the reference slice; 7^th^ degree optimum B-spline transformation), realigned (in other words corrected for motion to the mean position of each experimental set; 7^th^ degree optimum B-spline transformation) and co-registered to the structural images. Both functional and structural image sets were normalized to the MNI-152 space. Finally, the functional images were resampled to 2 × 2 × 2 mm resolution and spatially smoothed with a Gaussian kernel of 8 mm FWHM.

Two separate functional localizer runs (10.4 minutes long each, 20 sec epochs of famous faces, unfamiliar faces, chairs and Fourier randomized versions of faces, interleaved with 20 sec of blank periods, 2 Hz stimulus repetition rate; 300 ms exposure; 200 ms blank) served as basis for Regions of Interest (ROIs) detection. ROI creation was performed with MARSBAR 0.44 toolbox for SPM12^91^. The location of the FFA and OFA was determined individually, as an area responding more intensely to faces (famous and unfamiliar) than to chairs and Fourier randomized versions of faces (p < 0.0001_UNCORRECTED_). Both OFA and FFA could be identified bilaterally and reliably in 18 participants [average MNI coordinates (±SE): 1. FFA 40.9 (0.5), −46.9 (1.4), −20 (0.9) and −40.2 (0.7), −50.7 (0.9), −21.2 (0.6); average cluster size (±SE): 54 (2) and 53 (3) voxels; 2. OFA 41.8 (0.9), −77.3 (1.2), −8.8 (1.4) and −39.2 (1.0), −75.2 (1.8), −9.8 (1.8); average cluster size (±SE): 54 (3) and 54 (3) voxels, for right and left hemisphere, respectively].

The mean time series of all voxels within the ROIs (4 mm spheres around the MNI coordinate) were calculated and extracted from the event-related sessions using MARSBAR and custom made scripts. The convolution of the 3 experimental conditions (*Congruent*, *Incongruent*, *Unfamiliar*) with the canonical hemodynamic response function (HRF) of SPM12 served to define the predictors for a General Linear Model (GLM) analysis of the data.

### Statistical Analyses

Trials with unfamiliar face identities were considered as fillers, necessary for observing priming effects and were modelled, but excluded from the final analysis. Therefore, there are two conditions of interest: *Congruent* and *Incongruent*.

Repeated measures ANOVAs were performed for both behavioral and ROI data. Separate statistical analyses were conducted for the reaction times (RT) and accuracy of the recognition task with condition (2, *Congruent and Incongruent*; averaged across runs) as a factor. Similarly, separate repeated measures ANOVAs were performed for the FFA and OFA activity separately with hemisphere (2, Right and Left) and condition (2, *Congruent* and *Incongruent*) as factors. Post-hoc analyses were executed using Fisher LSD tests.

### Data Availability

The relevant datasets generated during the current study are available online from the OpenNeuro platform with the following DOI: 10.18112/openneuro.ds001357.v1.
